# 单倍型造血干细胞移植和HLA相合同胞供者造血干细胞移植治疗急性B淋巴细胞白血病的回顾性比较研究

**DOI:** 10.3760/cma.j.issn.0253-2727.2022.03.007

**Published:** 2022-03

**Authors:** 志东 王, 于谦 孙, 晨华 闫, 峰蓉 王, 晓冬 莫, 萌 吕, 晓甦 赵, 伟 韩, 欢 陈, 育红 陈, 昱 王, 兰平 许, 亚哲 王, 艳荣 刘, 翼飞 程, 晓辉 张, 开彦 刘, 晓军 黄, 英军 常

**Affiliations:** 北京大学人民医院血液科，北京大学血液病研究所，国家血液系统疾病临床医学研究中心，造血干细胞移植治疗血液病北京市重点实验室，北京 100044 Peking University People's Hospital & Peking University Institute of Hematology, National Clinical Research Center for Hematologic Disease, Beijing Key Laboratory of Hematopoietic Stem Cell Transplantation, Beijing 100044, China

**Keywords:** 单倍型造血干细胞移植, 人类白细胞抗原相合同胞供者移植, 微小残留病, 复发, Haploidentical stem cell transplantation, Human leukocyte antigen-matched sibling donor transplantation, Minimal residual disease, Relapse

## Abstract

**目的:**

探讨单倍型造血干细胞移植（haplo-HSCT）治疗移植前微小残留病（Pre-MRD）阳性急性B淋巴细胞白血病（B-ALL）是否较全相合同胞造血干细胞移植（MSDT）具有生存优势，以及该作用是否受Pre-MRD的影响。

**方法:**

对2009年6月至2018年6月在北京大学血液病研究所接受异基因造血干细胞移植（allo-HSCT）的998例移植前处于完全缓解（CR）的B-ALL患者进行回顾性分析，其中haplo-HSCT组788例、MSDT组210例，移植前用多参数流式细胞术（MFC）检测MRD水平。

**结果:**

①全部998例B-ALL患者中，997例获得持续的完全供者嵌合状态，移植后100 d中性粒细胞、血小板植入率分别为99.9％（997/998）、95.3％（951/998），Ⅱ～Ⅳ度急性移植物抗宿主病（GVHD）发生率为26.6％（95％*CI* 23.8％～29.4％），3年慢性GVHD累积发生率为49.1％（95％*CI* 45.7％～52.4％），移植后3年白血病累积复发率（CIR）为17.3％（95％*CI* 15.0％～19.7％），非复发死亡率（NRM）为13.8％（95％ *CI* 11.6％～16.0％），移植后3年无白血病生存（LFS）率、总生存（OS）率分别为69.1％（95％*CI* 66.1％～72.1％）、73.0％（95％*CI* 70.2％～75.8％）。②Pre-MRD阳性组（282例）患者移植后3年CIR高于Pre-MRD阴性组（716例）［31.6％（95％*CI* 25.8％～37.5％）对14.3％（95％*CI* 11.4％～17.2％），*P*<0.001］。③在Pre-MRD阳性组中，haplo-HSCT患者（219例）移植后3年CIR低于MSDT患者（63例）［27.2％（95％*CI* 21.0％～33.4％）对47.0％（95％*CI* 33.8％～60.2％），*P*＝0.002］。④全部998例患者按照Pre-MRD结果分为阴性组（716例）、<0.01％组（46例）、0.01％～<0.1％组（117组）、0.1％～<1％组（87例）、≥1％组（32例）；5组患者中，<0.01％组haplo-HSCT患者（40例）移植后3年CIR低于MSDT患者（6例）［10.0％（95％*CI* 0.4％～19.6％）对32.3％（95％*CI* 0％～69.9％），*P*＝0.017］，0.01％～<0.1％组haplo-HSCT患者（81例）移植后3年CIR也低于MSDT患者（36例）［20.4％（95％*CI* 10.4％～30.4％）对47.0％（95％*CI* 29.2％～64.8％），*P*＝0.004］；其他三组中，haplo-HSCT和MSDT患者移植后3年CIR差异无统计学意义。⑤在Pre-MRD<0.1％组（163例）中，haplo-HSCT患者（121例）移植后3年CIR低于MSDT患者（42例）［16.0％（95％*CI* 9.4％～22.7％）对40.5％（95％ *CI* 25.2％～55.8％），*P*<0.001］，3年LFS率、OS率均高于MSDT组［78.2％（95％*CI* 70.6％～85.8％）对47.6％（95％*CI* 32.2％～63.0％），*P*<0.001；80.5％（95％*CI* 73.1％～87.9％）对54.6％（95％*CI* 39.2％～70.0％），*P*<0.001］，两组3年NRM差异无统计学意义［5.8％（95％*CI* 1.6％～10.0％）对11.9％（95％*CI* 2.0％～21.8％），*P*＝0.188］。多因素分析显示，haplo-HSCT是Pre-MRD<0.1％组移植后低CIR（*HR*＝0.248，95％*CI* 0.131～0.472，*P*<0.001）、高LFS率（*HR*＝0.275，95％*CI* 0.157～0.483，*P*<0.001）和高OS率（*HR*＝0.286，95％*CI* 0.159～0.513，*P*<0.001）的独立影响因素。

**结论:**

haplo-HSCT治疗Pre-MRD<0.1％的B-ALL患者较MSDT具有生存优势。

## Abstract (en)

**Objective:**

To investigate whether haplotype hematopoietic stem cell transplantation（haplo-HSCT）is effective in the treatment of pre transplant minimal residual disease（Pre-MRD）positive acute B lymphoblastic leukemia（B-ALL）compared with HLA-matched sibling donor transplantation（MSDT）.

**Methods:**

A total of 998 patients with B-ALL in complete remission pre-HSCT who either received haplo-HSCT（*n*＝788）or underwent MSDT（*n*＝210）were retrospectively analyzed. The pre-transplantation leukemia burden was evaluated according to Pre-MRD determinedusing multiparameter flow cytometry（MFC）.

**Results:**

Of these patients, 997（99.9％）achieved sustained, full donor chimerism. The 100-day cumulative incidences of neutrophil engraftment, platelet engraftment, and grades Ⅱ-Ⅳ acute graft-versus-host disease（GVHD）were 99.9％（997/998）, 95.3％（951/998）, and 26.6％（95％*CI* 23.8％–29.4％）, respectively. The 3-year cumulative incidence of total chronic GVHD was 49.1％（95％*CI* 45.7％–52.4％）. The 3-year cumulative incidence of relapse（CIR）and non-relapse mortality（NRM）of the 998 cases were 17.3％（95％*CI* 15.0％–19.7％）and 13.8％（95％*CI* 11.6％–16.0％）, respectively. The 3-year probabilities of leukemia-free survival（LFS）and overall survival（OS）were 69.1％（95％*CI* 66.1％–72.1％）and 73.0％（95％*CI* 70.2％–75.8％）, respectively. In the total patient group, cases with positive Pre-MRD（*n*＝282）experienced significantly higher CIR than that of subjects with negative Pre-MRD［*n*＝716, 31.6％（95％*CI* 25.8％–37.5％）*vs* 14.3％（95％*CI* 11.4％–17.2％）, *P*<0.001］. For patients in the positive Pre-MRD subgroup, cases treated with haplo-HSCT（*n*＝219）had a lower 3-year CIR than that of cases who underwent MSDT［*n*＝63, 27.2％（95％*CI* 21.0％–33.4％）*vs* 47.0％（95％*CI* 33.8％–60.2％）, *P*＝0.002］. The total 998 cases were classified as five subgroups, including cases with negative Pre-MRD group（*n*＝716）, cases with Pre-MRD<0.01％ group（*n*＝46）, cases with Pre-MRD 0.01％–<0.1％ group（*n*＝117）, cases with Pre-MRD 0.1％–<1％ group（*n*＝87）, and cases with Pre-MRD≥1％ group（*n*＝32）. For subjects in the Pre-MRD<0.01％ group, haplo-HSCT（*n*＝40）had a lower CIR than that of MSDT［*n*＝6, 10.0％（95％*CI* 0.4％–19.6％）*vs* 32.3％（95％*CI* 0％–69.9％）, *P*＝0.017］. For patients in the Pre-MRD 0.01％–<0.1％ group, haplo-HSCT（*n*＝81）also had a lower 3-year CIR than that of MSDT［*n*＝36, 20.4％（95％*CI* 10.4％–30.4％）*vs* 47.0％（95％*CI* 29.2％–64.8％）, *P*＝0.004］. In the other three subgroups, the 3-year CIR was comparable between patients who underwent haplo-HSCT and those received MSDT. A subgroup analysis of patients with Pre-MRD<0.1％（*n*＝163）was performed, the results showed that cases received haplo-HSCT（*n*＝121）experienced lower 3-year CIR［16.0％（95％*CI* 9.4％–22.7％）*vs* 40.5％（95％*CI* 25.2％–55.8％）, *P*<0.001］, better 3-year LFS［78.2％（95％*CI* 70.6％–85.8％）*vs* 47.6％（95％*CI* 32.2％–63.0％）, *P*<0.001］and OS［80.5％（95％*CI* 73.1％–87.9％）*vs* 54.6％（95％*CI* 39.2％–70.0％）, *P*<0.001］than those of MSDT（*n*＝42）, but comparable in 3-year NRM［5.8％（95％*CI* 1.6％–10.0％）*vs* 11.9％（95％*CI* 2.0％–21.8％）, *P*＝0.188］. Multivariate analysis showed that haplo-HSCT was associated with lower CIR（*HR*＝0.248, 95％*CI* 0.131–0.472, *P*<0.001）, and superior LFS（*HR*＝0.275, 95％*CI* 0.157–0.483, *P*<0.001）and OS（*HR*＝0.286, 95％*CI* 0.159–0.513, *P*<0.001）.

**Conclusion:**

Haplo HSCT has a survival advantage over MSDT in the treatment of B-ALL patients with pre MRD<0.1％.

目前，单倍型造血干细胞移植（haplo-HSCT）已被广泛用于恶性血液病的治疗[1]。尽管haplo-HSCT治疗急性髓系白血病（AML）和急性淋巴细胞白血病（ALL）获得与同胞全相合造血干细胞移植（MSDT）相当的疗效，但由于haplo-HSCT后病毒感染发生率高等原因，MSDT仍是有移植适应证白血病患者的首选移植方式[2]–[4]。Nagler等[5]发现接受haplo-HSCT治疗的ALL患者移植后2年累积复发率（CIR）低于MSDT（*HR*＝0.66, *P*＝0.004），而haplo-HSCT后的高非复发死亡率（NRM）使低CIR未转化为生存优势[5]。我们前期研究发现，对于移植前微小残留病（Pre-MRD）阳性的AML和ALL患者而言，haplo-HSCT较MSDT具有生存优势[6]–[7]。此外，haplo-HSCT也在治疗恶性血液病[8]、难治/复发AML[9]以及霍奇金淋巴瘤[10]–[11]方面较MSDT具有生存优势。对接受haplo-HSCT或MSDT的ALL患者而言，Pre-MRD水平越高，移植后CIR也越高[12]–[14]。Zhao等[13]的研究提示haplo-HSCT有可能克服<0.1％的Pre-MRD对ALL患者移植预后的不良影响。尽管此前的研究提示haplo-HSCT治疗Pre-MRD阳性ALL患者较MSDT具有生存优势，但作者未进行亚组分析[6]。根据此前的研究[5]–[7],[14]–[16]，我们推测对于特定Pre-MRD水平的ALL患者而言，haplo-HSCT才能较MSDT显示出优势。为此，我们拟通过扩大患者数量来探讨haplo-HSCT是否较MSDT在治疗急性B淋巴细胞白血病（B-ALL）具有生存优势以及该优势是否受到Pre-MRD水平的影响。

## 病例与方法

1. 研究对象：本研究回顾性纳入了2009年6月至2018年6月在北京大学血液病研究所接受allo-HSCT、移植前处于血液学缓解（HCR）状态的998例B-ALL患者。供者选择[3],[6]：HLA相合同胞供者（MSD）作为首选；如果没有MSD，且无合适的无关供者（HLA-A、B、C、DR和DQ位点>8/10或HLA-A、B、DR位点>5/6），则选择单倍型供者。所有患者获得HCR后行2疗程巩固治疗后接受移植[3],[6]，本研究经北京大学人民医院伦理委员会批准，所有供、患者均签署知情同意书。

2. 移植方案：造血干细胞动员、移植物采集和回输、预处理方案和移植物抗宿主病（GVHD）预防及移植后MRD干预参见文献[6],[13]。

3. MRD检测：MRD检测的时间点包括移植前（预处理前4周）、移植后1、2、3、4.5、6、9和12个月，1年后每6个月评估1次MRD[6],[13]。检测方法：①应用白血病相关免疫表型和/或与正常骨髓细胞表型相鉴别的方法借助MFC评估MRD；②借助实时定量聚合酶链反应技术（Q-PCR）测定WT1或BCR/ABL等泛白血病和（或）白血病特异基因评估MRD。具体参见文献[6],[13]。

4. 随访：中位随访时间为44（0.4～135）个月。主要观察终点是白血病CIR；次要观察终点包括造血植入、Ⅱ～Ⅳ度急性GVHD、慢性GVHD、NRM、无白血病生存（LFS）和总生存（OS）等。主要观察终点和次要观察终点的定义见文献[6],[13]。

5. 统计学处理：计数和计量资料用频率和中位数等进行描述统计。MSDT和haplo-HSCT组间供受者特征比较采用卡方检验或Fisher精确检验（分类变量）以及*t*检验或非参数检验。造血植入、急性和慢性GVHD、LFS以及OS等采用Kaplan-Meier进行描述和计算。单因素和多因素分析采用Cox模型进行统计，*P*<0.1的变量被纳入多因素分析。采用SPSS 19.0软件进行数据分析。NRM和CIR采用R软件进行竞争风险分析。

## 结果

1. 总体B-ALL患者的一般资料和移植结果：998例患者中男558例、女440例，中位年龄26（2～63）岁，其中Ph染色体阳性患者346例（34.7％）、移植前疾病状态≥第2次完全缓解（CR2）149例（14.9％）（表1）。所有患者均接受清髓预处理。全部998例中997例获得中性粒细胞植入，中位植入时间为移植后13（9～66）d，移植后100 d植入率为99.9％（997/998）；中位血小板植入时间为15（4～418）d，移植后100 d植入率为95.3％（951/998）。移植后100 d Ⅱ～Ⅳ度急性GVHD发生率为26.6％（95％*CI* 23.8％～29.4％）。中位随访44（0.4～135）个月，3年慢性GVHD累积发生率为49.1％（95％*CI* 45.7％～52.4％），CIR为17.3％（95％*CI* 15.0％～19.7％），NRM为13.8％（95％*CI* 11.6％～16.0％）；3年LFS、OS率分别为69.1％（95％*CI* 66.1％～72.1％）、73.0％（95％*CI* 70.2％～75.8％）。

2. 不同Pre-MRD水平对B-ALL患者预后的影响：998例患者分为Pre-MRD阴性组（716例）和阳性组（282例），结果显示阳性组患者移植后的CIR显著高于阴性组［31.6％（95％*CI* 25.8％～37.5％）对14.3％（95％*CI* 11.4％～17.2％），*P*<0.001］。Pre-MRD结果分为阴性组（716例）、<0.01％组（46例）、0.01％～<0.1％组（117组）、0.1％～<1％组（87例）、≥1％组（32例）；5组患者中，<0.01％组中haplo-HSCT患者（40例）移植后3年CIR低于MSDT患者（6例）［10.0％（95％*CI* 0.4％～19.6％）对32.3％（95％*CI* 0％～69.9％），*P*＝0.017］，0.01％～<0.1％组haplo-HSCT患者（81例）移植后3年CIR也低于MSDT患者（36例）［20.4％（95％*CI* 10.4％～30.4％）对47.0％（95％*CI* 29.2％～64.8％），*P*＝0.004］；其他三组中，haplo-HSCT和MSDT患者移植后3年CIR差异无统计学意义。提示，haplo-HSCT有可能克服<0.1％的Pre-MRD水平对移植预后的不良影响。

3. haplo-HSCT和MSDT对Pre-MRD阳性B-ALL患者预后的影响：998例患者被分为Pre-MRD阴性接受haplo-HSCT（B1组）和MSDT组（B2组）以及Pre-MRD阳性接受haplo-HSCT（B3组）和MSDT组（B4组），结果显示B1组和B2组患者移植后3年CIR差异无统计学意义［14.5％（95％*CI* 11.3％～17.9％）对12.9％（95％*CI* 7.1％～18.7％），*P*＝0.944］，B3组移植后3年CIR显著低于B4组［27.2％（95％*CI* 21.0％～33.4％）对47.0％（95％*CI* 33.8％～60.2％），*P*＝0.002］，二者显著高于B1和B2组（*P*<0.01）。在Pre-MRD<0.01％和0.01％～<0.1％组，haplo-HSCT患者移植后3年CIR低于MSDT患者［Pre-MRD<0.01％组：10.0％（95％*CI* 0.4％～19.6％）对32.3％（95％*CI* 0％～69.9％），*P*＝0.017；Pre-MRD 0.01％～<0.1％组：20.4％（95％*CI* 10.4％～30.4％）对47.0％（95％*CI* 29.2％～64.8％），*P*＝0.004］。

4. haplo-HSCT和MSDT对Pre-MRD<0.1％ B-ALL患者预后的影响：依据移植模式将163例对Pre-MRD<0.1％患者分为haplo-HSCT组（121例）和MSDT组（42例），haplo-HSCT组年龄低于MSDT组（*P*＝0.002）、接受G-CSF动员的外周血采集物（G-PB），患者比例低于MSDT组（*P*＝0.004）；此外，haplo-HSCT组移植后接受MRD指导干预的患者比例低于MSDT组（*P*＝0.011），详见表1。

**表1:** 接受allo-HSCT的998例急性B淋巴细胞白血病（B-ALL）及163例移植前微小残留病（MRD）<0.1％患者的临床资料

临床特征	总体（998例）	移植前MRD < 0.1％			
		haplo-HSCT（121例）	MSDT（42例）	统计量	*P*值
年龄［岁，*M*（范围）］	26（2～63）	26（4～58）	37（8～60）	−3.331	0.002
性别［例（％）］				0.080	0.778
男	558（55.9）	69（57.0）	25（59.5）		
女	440（44.1）	52（43.0）	17（40.5）		
Ph染色体［例（％）］				1.218	0.270
阳性	346（34.7）	58（47.9）	16（38.1）		
阴性	652（65.3）	63（52.1）	26（61.9）		
移植前疾病状态［例（％）］				1.661	0.286
CR1	849（85.1）	103（85.1）	39（92.9）		
≥CR2	149（14.9）	18（14.9）	3（7.1）		
移植前病程［月，*M*（范围）］	6.5（2.5～192.0）	6.0（3.0～69.5）	6.0（3.0～24.5）	−0.794	0.492
供受者性别组合［例（％）］				7.735	0.052
女供男	195（19.5）	25（20.7）	15（35.7）		
女供女	144（14.4）	15（12.4）	9（21.4）		
男供女	290（29.1）	37（30.6）	7（16.7）		
男供男	369（36.9）	44（36.4）	11（26.2）		
HLA位点不合［例（％）］				169.745	<0.001
0个位点	217（21.7）	2（1.7）	42（100）		
1个位点	27（2.7）	0	0		
2个位点	140（14.0）	18（14.9）	0		
3个位点	614（61.5）	101（83.5）	0		
供受者血型组合［例（％）］				3.638	0.303
相合	554（55.5）	68（56.2）	24（57.1）		
主要不合	202（20.2）	31（25.6）	6（14.3）		
次要不合	195（19.5）	17（14.0）	10（23.8）		
主要不合+次要不合	47（4.7）	5（4.1）	2（4.8）		
供受者关系［例（％）］				75.314	<0.001
父母供子女	447（44.8）	57（47.1）	0		
同胞供同胞	426（42.7）	38（31.4）	42（100）		
子女供父母	107（10.7）	24（19.8）	0		
其他	18（1.8）	2（1.7）	0		
移植物［例（％）］				11.143	0.004
G-PB+G-BM	975（97.7）	121（100）	38（90.5）		
G-PB	23（2.3）	0	4（9.5）		
移植物有核细胞［×10^8^/kg，*M*（范围）］	8.17（2.53～20.12）	7.91（5.58～15.67）	7.73（5.34～11.86）	−1.368	0.138
移植物CD34^+^细胞［×10^6^/kg，*M*（范围）］	2.43（0.38～12.66）	2.25（0.84～8.07）	2.45（0.90～6.39）	−0.918	0.404
移植后接受MRD指导干预［例（％）］	163（16.3）	32（26.4）	21（50.0）	6.463	0.011
仅减停免疫抑制剂	36（3.6）	4（3.3）	3（7.1）		
干扰素	57（5.7）	13（10.7）	7（16.7）		
DLI	34（3.4）	5（4.1）	5（11.9）		
靶向药物	36（3.6）	10（8.3）	6（14.3）		

注：allo-HSCT：异基因造血干细胞移植；haplo-HSCT：单倍型造血干细胞移植；MSDT：同胞全相合造血干细胞移植；CR1、CR2分别为第1、2次完全缓解；G-PB：粒细胞集落刺激因子（G-CSF）动员的外周血采集物；G-BM：G-CSF动员的骨髓采集物；DLI：供者淋巴细胞输注

haplo-HSCT组的血小板植入率显著低于MSDT组［98％（119/121）对100％（42/42），*P*<0.001］；haplo-HSCT组移植后3年CIR低于MSDT组［16.0％（95％*CI* 9.4％～22.7％）对40.5％（95％*CI* 25.2％～55.8％），*P*<0.001］，LFS和OS均显著高于MSDT组［78.2％（95％*CI* 70.6％～85.8％）对47.6％（95％*CI* 32.2％～63.0％），*P*<0.001；80.5％（95％*CI* 73.1％～87.9％）对54.6％（95％*CI* 39.2％～70.0％），*P*<0.001］（表2、图1）。单因素分析显示，影响CIR的变量有移植前CR状态（*P*＝0.063）、移植模式（*P*<0.001）和发生慢性GVHD（*P*＝0.008）；影响NRM的变量有患者性别（*P*＝0.083）和发生Ⅱ～Ⅳ度急性GVHD（*P*＝0.044）；影响LFS的变量有移植前CR状态（*P*＝0.055）、移植模式（*P*<0.001）和发生慢性GVHD（*P*＝0.002）；影响OS的因素有移植前CR状态（*P*＝0.020）和移植模式（*P*<0.001）。多因素分析显示，移植模式是移植后CIR（*HR*＝0.248，*P*<0.001）、LFS（*HR*＝0.275，*P*<0.001）和OS（*HR*＝0.286，*P*<0.001）的独立影响因素（表3、图1）。病例配对分析显示，haplo-HSCT组较MSDT组具有更低的CIR［13.8％（95％*CI* 6.0％～21.6％）对45.0％（95％*CI* 28.6％～61.4％），*P*<0.001］、更高的LFS［80.9％（95％*CI* 72.3％～89.4％）对47.6％（95％*CI* 32.2％～63.0％），*P*<0.001］和OS［84.2％（95％*CI* 76.2％～92.2％）对54.6％（95％*CI* 39.2％～70.0％），*P*<0.001］，多因素分析显移植模式是移植后CIR（*HR*＝0.263，*P*<0.001）、LFS（*HR*＝0.299，*P*<0.001）和OS（*HR*＝0.263，*P*<0.001）的独立影响因素（表4）。

**表2:** haplo-HSCT和MSDT治疗移植前微小残留病（MRD）<0.1％急性B淋巴细胞白血病（B-ALL）患者的预后

临床特征	总体（163例）	haplo-HSCT（121例）	MSDT（42例）	*P*值^a^
+100 d中性粒细胞植入率	100％	100％	100％	1.000
+100 d血小板植入率	95.7％（95％*CI* 92.5％~98.9％）	94.2％（95％*CI* 90.0％~98.4％）	100％	<0.001
+100 d Ⅱ~Ⅳ度急性GVHD发生率	24.7％（95％*CI* 17.9％~31.3％）	26.5％（95％*CI* 18.5％~34.5％）	19.5％（95％*CI* 7.1％~31.9％）	0.272
3年累积慢性GVHD发生率	51.1％（95％*CI* 42.5％~59.7％）	49.0％（95％*CI* 39.2％~58.8％）	58.7％（95％*CI* 41.3％~76.1％）	0.491
3年累积复发率	22.4％（95％*CI* 15.9％~28.9％）	16.0％（95％*CI* 9.4％~22.7％）	40.5％（95％*CI* 25.2％~55.8％）	<0.001
3年非复发死亡率	7.4％（95％*CI* 3.3％~11.4％）	5.8％（95％*CI* 1.6％~10.0％）	11.9％（95％*CI* 2.0％~21.8％）	0.188
3年无病生存率	70.2％（95％*CI* 63.0％~77.4％）	78.2％（95％*CI* 70.6％~85.8％）	47.6％（95％*CI* 32.2％~63.0％）	<0.001
3年总生存率	73.7％（95％*CI* 67.7％~80.7％）	80.5％（95％*CI* 73.1％~87.9％）	54.6％（95％*CI* 39.2％~70.0％）	<0.001

注：haplo-HSCT：单倍型造血干细胞移植；MSDT：同胞全相合造血干细胞移植；GVHD：移植物抗宿主病。^a^ MSDT组与haplo-HSCT组比较

**图1:**
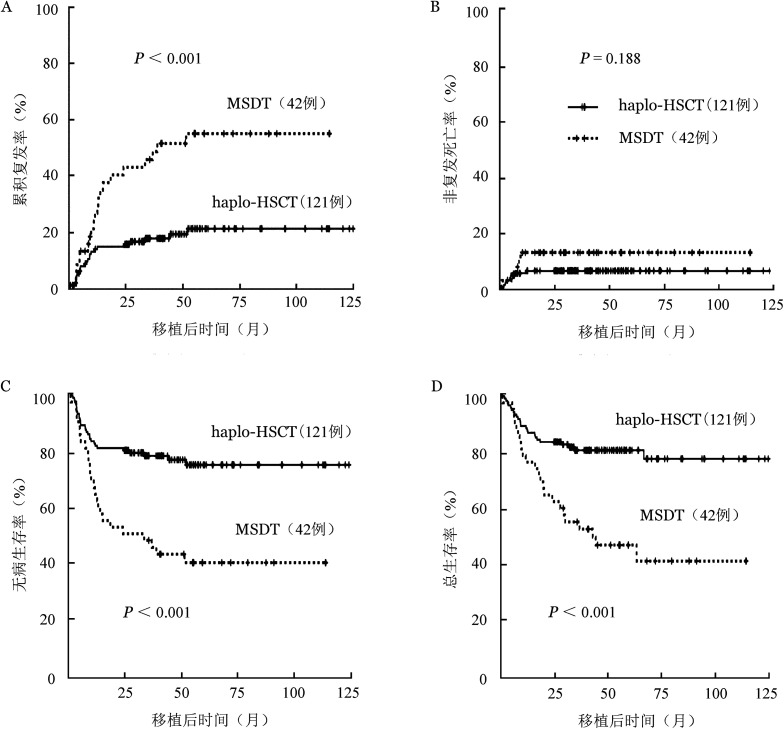
移植模式对移植前微小残留病<0.1％急性B淋巴细胞白血病患者造血干细胞移植预后的影响 haplo-HSCT：单倍型造血干细胞移植；MSCT：同胞全相合造血干细胞移植；A：累积复发；B：非复发死亡；C：无病生存；D：总生存

**表3:** 移植前微小残留病（MRD）<0.1％急性B细胞淋巴细胞白血病（B-ALL）患者预后影响因素分析

影响因素	单因素分析		多因素分析	
	*HR*（95％ *CI*）	*P*值	*HR*（95％ *CI*）	*P*值
复发				
移植前疾病状态（≥CR2，CR1）	2.083（0.961～4.512）	0.063	3.031（1.351～6.800）	0.007
移植模式（haplo-HSCT，MSDT）	0.314（0.170～0.581）	<0.001	0.248（0.131～0.472）	<0.001
慢性GVHD（发生，未发生）	0.402（0.205～0.788）	0.008	0.345（0.174～0.682）	0.002
非复发死亡				
性别（男，女）	0.261（0.057～1.192）	0.083		
Ⅱ～Ⅳ度急性GVHD（发生，未发生）	3.209（1.034～9.953）	0.044	3.209（1.034～9.953）	0.044
无病生存				
移植前疾病状态（≥CR2，CR1）	1.960（0.984～3.903）	0.055	2.741（1.339～5.611）	0.006
移植模式（haplo-HSCT，MSDT）	0.342（0.199～0.586）	<0.001	0.275（0.157～0.483）	<0.001
慢性GVHD（发生，未发生）	0.391（0.215～0.712）	0.002	0.342（0.187～0.627）	0.001
总生存				
移植前疾病状态（≥CR2，CR1）	2.301（1.143～4.631）	0.020	2.961（1.448～6.053）	0.003
移植模式（haplo-HSCT，MSDT）	0.325（0.183～0.575）	<0.001	0.286（0.159～0.513）	<0.001

注：CR1、CR2分别为第1、2次完全缓解；haplo-HSCT：单倍型造血干细胞移植；MSDT：同胞全相合造血干细胞移植；GVHD：移植物抗宿主病

**表4:** 移植前MRD<0.1％的B-ALL患者预后影响的单因素与多因素分析（MSDT与haplo-HSCT患者配比1∶2）

影响因素	单因素分析		多因素分析	
	*HR*（95％ *CI*）	*P*值	*HR*（95％ *CI）*	*P*值
复发				
移植模式（haplo-HSCT，MSDT）	0.271（0.135～0.546）	<0.001	0.263（0.130～0.530）	<0.001
慢性GVHD（发生，未发生）	0.494（0.243～1.005）	0.052	0.468（0.230～0.953）	0.036
非复发死亡				
性别（男，女）	0.150（0.019～1.181）	0.072	0.150（0.019～1.181）	0.072
无病生存				
移植模式（haplo-HSCT，MSDT）	0.308（0.168～0.566）	<0.001	0.299（0.163～0.549）	<0.001
慢性GVHD（发生，未发生）	0.473（0.252～0.886）	0.019	0.451（0.241～0.847）	0.013
总生存				
移植模式（haplo-HSCT，MSDT）	0.263（0.135～0.512）	<0.001	0.263（0.135～0.512）	<0.001

注：MRD：微小残留病；B-ALL：急性B淋巴细胞白血病；CR：完全缓解；haplo-HSCT：单倍型相合造血干细胞移植；MSDT：同胞全相合造血干细胞移植；GVHD：移植物抗宿主病

## 讨论

本研究结果证实，haplo-HSCT治疗Pre-MRD阳性的B-ALL患者较MSDT具有生存优势，与此前其他学者[5],[15]和我们的研究结果[6]–[7],[18],[20]一致。更重要的是，我们还发现haplo-HSCT较MSDT的生存优势仅限于Pre-MRD<0.1％的B-ALL患者；在Pre-MRD阴性或≥0.1％的患者中，haplo-HSCT并未显示出较MSDT具有生存优势。本研究为以前的研究[5]–[7],[16]–[20]增添了新的内容，即对于移植前特定白血病负荷的B-ALL亚组人群，haplo-HSCT才能显示出较MSDT具有生存优势。

近年来，越来越多的研究显示haplo-HSCT治疗恶性血液病较MSDT具有更强的移植物抗白血病（GVL）作用[5]–[7],[18]–[20]，然而，并非所有的报道都显示haplo-HSCT较强的GVL作用可转化为生存优势[5],[20]；这可能与下列因素有关：①haplo-HSCT后NRM显著高于MSDT[5]。②多数研究报道的是恶性血液病患者总体人群，包括AML、ALL和骨髓增生异常综合征等[7]–[8]；Luo等[8]发现对于接受allo-HSCT的恶性血液病患者而言，haplo-HSCT后5年CIR显著低于MSDT（14.2％对34％，*P*＝0.008），但两组5年LFS和OS率并无统计学差异。因此，如何从恶性血液病患者总体人群筛选出能从生存上获益于haplo-HSCT更强GVL作用的亚组人群就显得尤为重要。本研究和我们此前的研究[6]–[7],[18],[20]均显示作为生物学标志的Pre-MRD不仅可用于筛选获益于单倍型供者更强GVL作用的ALL患者，而且还可用于筛选AML患者。

与此前研究[6],[18],[20]不同的是，我们在本研究中确定了Pre-MRD<0.1％的B-ALL患者是生存上可获益于haplo-HSCT更强GVL作用的亚组人群；在该亚组患者中，尽管接受MSDT的患者移植后复发干预的比例显著高于haplo-HSCT组，但haplo-HSCT组患者仍较MSDT组患者复发率低、生存好；haplo-HSCT较MSDT更强的GVL效应可能得益于单倍型相合T细胞较同胞相合T细胞具有更强的同种反应性，这在临床上表现为haplo-HSCT移植后的急性GVHD发生率显著高于MSDT[3]。我们的结果提示haplo-HSCT的GVL作用并非无限强于HLA相合同胞供者，对于Pre-MRD阴性患者，二类供者都具有足够的GVL作用；而Pre-MRD阴性患者接受两类移植后10％左右的CIR可能与MRD检测方法所致的假阴性结果有关[21]。对于Pre-MRD≥0.1％的B-ALL人群而言，haplo-HSCT和MSDT两种移植模式都不能克服移植前白血病负荷对预后的不良影响；该组人群中部分患者接受两类移植后均未复发一方面可能与MRD检测方法导致的假阳性结果有关[21]，另一方面可能与白血病的生物学特性有关[22]，即haplo-HSCT或MSDT仍对于部分Pre-MRD≥0.1％的B-ALL患者体内的白血病细胞具有GVL作用。此外，移植后白血病细胞能否被清除、疾病获得治愈，取决于白血病细胞和免疫细胞（包括T细胞和自然杀伤细胞）之间的强弱对比，Guo等[18]最近研究发现，免疫细胞和移植前白血病负荷的数量对比可能与其发挥GVL作用密切相关，这也部分解释了为什么对于移植前特定白血病符合范围内的患者（Pre-MRD<1％），haplo-HSCT才能显示出较MSDT更强的GVL作用。当然，明确的机制尚需深入研究。

本研究为回顾性研究，存在如下局限性：首先，我们的分析仅仅是限定在B-ALL人群，因此，有必要就T-ALL人群进行研究，以确定移植前白血病负荷是否影响haplo-HSCT或MSDT的GVL作用；其次，本研究为单中心分析，应该开展前瞻性、多中心研究以探讨哪个亚组的ALL患者人群可获益于haplo-HSCT更强的GVL效应[23]–[25]；再次，我们在本研究中借助MFC方法来确定Pre-MRD的阴性和阳性，而采用Q-PCR方法检测MRD的敏感性和特异性优于MFC[17],[26]，因此，对于具有特定融合基因（例如BCR/ABL）的患者应该探讨Q-PCR方法检测Pre-MRD在haplo-HSCT和MSDT对ALL患者预后影响中的价值。

本研究结果显示，对于Pre-MRD<1％的B-ALL患者，haplo-HSCT模式在生存方面优于MSDT，在有经验的移植中心，可选择haplo-HSCT模式治疗Pre-MRD<1％的B-ALL患者。
